# Enhanced Wear Performance of Cu-Carbon Nanotubes Composite Coatings Prepared by Jet Electrodeposition

**DOI:** 10.3390/ma12030392

**Published:** 2019-01-27

**Authors:** Dongdong Ning, Ao Zhang, Hui Wu

**Affiliations:** State Key Laboratory of New Ceramics and Fine Processing, School of Materials Science and Engineering, Tsinghua University, Beijing 100084, China; zhanga15@mails.tsinghua.edu.cn

**Keywords:** jet electrodeposition, Cu-CNTs composite coatings, wear performance

## Abstract

Cu-carbon nanotubes (CNTs) composite coatings with high CNT content and uniformly distributed CNTs were successfully prepared via jet electrodeposition. Pristine CNTs, without any treatment like acid functionalization, were used. Anionic surfactant sodium dodecyl sulfate (SDS) was used to increase the wettability of the CNTs and improve the content of incorporated CNTs. With an appropriate SDS concentration (300 mg/L) in the electrolyte, the incorporated CNT content is as high as 2.84 wt %, much higher than the values reported using conventional electrodeposition (0.42–1.05 wt %). The high-content CNTs were uniformly distributed in the composite coating. The surface morphology of this composite coating (2.84 wt % CNTs) was flat due to the uniform electric field in jet electrodeposition. In the wear test a with load of 1 N and sliding speed of 0.02 m/s, the wear rate of this composite coating was 1.3 × 10^−2^ mg/Nm, 85.4% lower than that of pure Cu. The enhanced wear performance of Cu-CNTs composite coatings can be attributed to high CNT content and flat surface morphology.

## 1. Introduction

Metal matrix composites with enhanced second phase in metal matrix show desirable metal properties and significantly improved mechanical properties [1,2,3]. There are many routes to fabricating metal matrix composites including casting, thermal sparing, and mechanical alloying. These methods have some drawbacks, such as severe reaction between the second phase and metal matrix, high temperature conditions, and high cost. In contrast with the above methods, electrodeposition has been widely used to produce metal matrix composites due to its operation in low temperature conditions and low cost. The second phase, including SiC [4,5,6], TiO_2_ [7,8,9,10], carbon nanotubes (CNTs) [11,12,13,14,15,16], and Poly tetra fluoroethylene (PTFE) [17], have been successfully co-deposited into a metal matrix using electrodeposition.

CNTs exhibit desirable electrical conductivity, thermal conductivity, and mechanical properties (tensile strength 1 TPa and tensile strength 63 GPa) [18,19], making them suitable as a reinforced phase in the metal matrix. Cu-CNTs composite coatings with high thermal conductivity, enhanced strength, and wear resistance have been extensively studied [11,12,13,14]. Conventional electrodeposition is usually used to prepare Cu-CNTs composite coatings. Cu-CNTs films fabricated by electrodeposition under ultrasonic filed showed 36% enhanced microhardness compared to pure Cu [20]. Cu-0.42 wt % Mutil-walled carbon nanotubes (MWCNTs) prepared by electrodeposition have a high thermal conductivity of 355 W m^−1^ K^−1^ [16]. The resistivity of Cu-MWCNTs (0.55 wt %) composite film is 44% lower than that of electroformed pure Cu films [21]. Cu matrix reinforced by 0.94 wt % CNTs showed 300% enhanced tensile stress compared to pure Cu [22]. Cu-CNTs composite with CNT content as high as 1.05 wt % was prepared by electrodeposition with polyacrylic acid added as the dispersant [15]. Periodic pulse reverse electrodeposition was used to prepare Cu-CNTs composite thin films. CNT agglomerations can be effectively avoided when nano-diamond is used as a dispersing agent [23]. In order to increase CNT hydrophilicity and improve incorporated CNT content, CNTs were immersed in strong acid to achieve functionalization before electrodeposition. The strong acid treatment process was complex and the effect was limited [13,22,23,24,25,26]. A simple and feasible method is still required to fabricate Cu-CNTs composite coatings with high CNT content and uniformly distributed CNTs.

In contrast to conventional electrodeposition, more of the second phase can reach the cathode surface due to the high speed flow of the electrolyte in jet electrodeposition. Therefore, the probabilities of the second phase being captured by a metal matrix increase and the incorporated content can be significantly increased. The scouring of high-speed electrolyte flow can effectively reduce second phase agglomeration in the deposition layer by washing the agglomerated phase away when them are weakly adsorbed on the cathode surface. Jet electrodeposition has been successfully applied to prepare Cu-Al_2_O_3_ and Ni-SiO_2_ composite coatings [27,28,29,30,31].

To the best of our knowledge, few papers have been published about Cu-CNTs composite coatings prepared by jet electrodeposition. In this work, a simple and feasible jet electrodeposition method was used to prepare Cu-CNTs composite coatings with high CNT content and uniform CNT distribution. Without any treatment, like acid functionalization and high-energy ball milling, the CNTs were directly added to the electrolyte. Anionic surfactant sodium dodecyl sulfate (SDS) was used to improve the wettability of the CNTs. With appropriate jet velocity and deposition rate, the agglomerated CNTs were almost non-existent in the composite layer. The surface morphology of this composite coating was quite flat due to the uniform electric field in jet electrodeposition. The incorporated CNTs can be as high as 2.84 wt %, much higher than that of Cu-CNTs composite coatings prepared by conventional electrodeposition (0.42–1.05 wt %) [14,15,16,21]. This composite coating showed enhanced wear performance due to the high content of self-lubrication CNTs and flat surface morphology.

## 2. Experimental Procedures

The electrolyte included copper sulfate (200 g/L, Sinopharm Chemical Reagent Co., Ltd., Shanghai, China) and sulfuric acid (60 g/L, Sinopharm Chemical Reagent Co., Ltd., Shanghai, China). Multi-walled CNTs (10–20 nm outer diameter, 2 μm length, >97% purity) were provided by Shenzhen Nanotech Port Co., Ltd. (Shenzhen, China) and 2 g/L CNTs were directly added to the electrolyte. The SDS concentration varied from 100 mg/L to 500 mg/L. The mixture of electrolytes was agitated by magnetic stirring for 3 h to uniformly disperse CNTs in the electrolyte. An anti-corrosion pump with controlled flow was used in the experiment (39-05, DingFeng Electronics Co., Ltd., Dongguan, China). The jet rate of the electrolyte was set to 1.0 m/s in order to obtain high CNT content. A pure copper pipe (25 mm length, 5 mm inner diameter) was set as a soluble anode. The flow rate was set to 1.16 L/min to ensure the jet rate of 1.0 m/s. The stainless steel (10 mm width, 20 mm length) was used as the cathode substrate. By cutting a 5 mm hole into insulated 3M tape (Minnesota Mining and Manufacturing Co., Maplewood, MN, USA, a 5-mm-diameter circle of the stainless steel was exposed to the electrolyte and other parts were covered by 3M tape. The size of the cathode (5 mm diameter) was the same as the anode nozzle (5 mm inner diameter) for the purpose of producing composite coatings with uniform thickness. The distance between the copper anode and stainless steel cathode electrode was 10 mm. The current density was set to 5 A/dm^2^ and maintained for 1 h to produce a 70-μm-thick composite coating. The size of the obtained Cu-CNTs composite coating was a circle with a diameter of 5 mm.

Before the wear test, the 3M tape was peeled off the stainless substrate. The weight of stainless steel substrate and Cu-CNTs composite coatings was measured by an electronic balance (ME36S, Sartorius, Gottingen, Germany) and the total weight was recorded as m_1_. Then, the stainless steel covered by Cu-CNT composite coatings (5 mm diameter) was stuck to the stage of the friction instrument (UMT-3, Bruker, Karlsruhe, Germany) using 3M double-sided tape. A GCr15 ball transducer (G10, Shanghai precision bearing co. LTD, Shanghai, China) was used as the counter body and the friction coefficient was recorded by a sensitive transducer (DFM-0.5, CETR, Allentown, PA, USA). Dry ball wear tests were performed on the surface of the composite coatings with a constant applied load of 1 N and sliding speed of 0.02 m/s. The total wear time was 20 min. After the wear test, the stainless steel was separated from the stage of the friction instrument. The weights of the stainless steel substrate and Cu-CNTs composite coatings after the wear test were measured using an electronic balance (ME36S, Sartorius, Gottingen, Germany) and the total weight was recorded as m_2_. The weight loss of the composite coatings (m_1_–m_2_) during the wear test was finally obtained.

The surface morphology of the composite coatings on the micrometer scale was observed using an optical microscope (BX35, OLYMPUS, Tokyo, Japan). The morphology of the composite coatings on the nanoscale was measured by scanning electron microscopy (SEM; MERLIN VP Compact, ZEISS, Oberkochen, Germany). X-ray diffraction (XRD) measurements were recorded using a D/max-2500 diffraction instrument (Rigaku, Tokyo, Japan) with Cu K-alpha radiation (40 kV, 40 mA). The microhardness of the Cu-CNTs composite coatings was measured using a Vickers microhardness measuring device (XHV-1000, SCTMC, Shanghai, China) with a load of 50 g and duration of 10 s. Different locations on the flat area of the composite coatings were selected to measure the microhardness. Each sample was measured 5 times and the average value was computed. The CNT content in the composite coatings was measured by inductively coupled plasma-optical emission spectrometry (Vista-MPX, Varian, Palo Alto, CA, USA). The weight loss of the Cu-CNTs composite coatings was measured by a high resolution (0.001 mg) electronic balance (ME36S, Sartorius, Gottingen, Germany).

## 3. Results and Discussion

**Figure 1:**
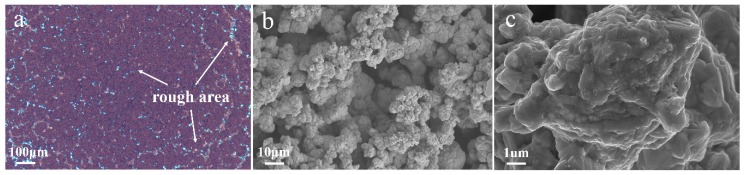
(**a**) Optical microscope image and (**b**,**c**) surface Scanning electron microscope (SEM) micrograph of Cu-CNTs composite (without SDS) by jet electrodeposition.

Table 1 shows the Zeta potential and CNT content with different SDS concentrations in the electrolyte. The optical microscopy image of this composite coating (Figure 1a) shows that a large number of rough areas existed on the composite surface, which is consistent with the SEM micrographs (Figure 1b,c). The low zeta potential value shows that the CNTs easily agglomerate. The agglomerated CNTs adsorbed on the cathode surface can block local metal crystal growth, which results in a very rough surface of the composite coating, as shown in Figure 1. 

With increasing SDS concentration, the more negative the Zeta potential of the CNTs, the better the dispersibility of the CNTs. The more negative the Zeta potential of the CNTs, the larger the repulsive force between the CNTs, which effectively reduces CNTs agglomeration. The CNT content was as high as 2.84% when the optimum SDS was added to the electrolyte (300 mg/L). Beyond the optimum concentration (500 mg/L), the CNT content decreased slightly. Excessive SDS forming micelles and adsorbing on the cathode will hamper the CNTs co-deposition process [10,32]. A greater repulsive force between the cathode and more negatively charged CNTs also hamper the process of CNTs co-deposition. As such, the CNT content was lower in the composite coatings with high SDS concentration.

**Figure 2:**
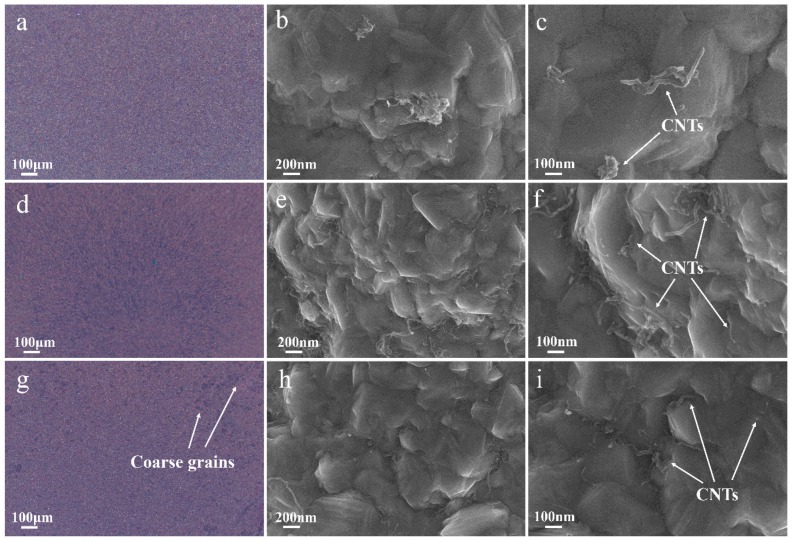
The effect of different concentrations of SDS on the surface morphology of the Cu-CNTs composite coatings. (**a**–**c**) 100 m/L SDS, (**d**–**f**) 300 mg/L SDS, and (**h**–**j**) 500 mg/L SDS.

The influence of SDS concentration on the morphology of Cu-CNTs composite coatings is shown in Figure 2. When the SDS concentration is 100 mg/L, the optical microscope image (Figure 2a) demonstrated that the surface of this composite coating is quite flat on the micrometer scale. As shown in Figure 2b,c, a small amount of dispersed CNTs and some agglomerated CNTs can be found on the surface of this composite coating. With low SDS concentration, the Zeta potential of CNTs slightly decreased, which means the CNTs were not well dispersed and a certain amount of CNTs were agglomerated in the electrolyte. According to Guglielmi’s two-step adsorption model [33], these agglomerated CNTs can be easily washed away by jet flow when they are weakly adsorbed on the cathode surface, which results in a low CNTs content in the composite layer. When the optimum SDS concentration (300 mg/L) was added to the electrolyte, the surface morphology on the micrometer scale was also quite flat (Figure 2d). As shown in Figure 2e,f, a large amount of uniformly distributed CNTs were found on the surface of this composite coating. The CNTs were uniformly dispersed when the optimum SDS concentration was added to the electrolyte. These uniformly dispersed CNTs were captured by the Cu matrix during the deposition process, which resulted in a high CNTs content and uniformly distributed CNTs in the composite layer. Beyond the optimum SDS concentration, coarse grains were found on the surface (Figure 2g). At high SDS concentration (500 mg/L), excessive SDS formed micelles adsorbed on the cathode surface, which hampered CNTs co-deposition and formed coarse grains [32]. The highly dispersed CNTs on the surface are shown in Figure 2h,i. With high SDS concentration, the zeta potential of CNTs became more negative. A higher repulsive force between the cathode and the more negatively charged CNTs also hamper the process of CNTs co-deposition. These SEM micrograph results are highly consistent with the CNTs content results shown in Table 1. By adding pristine CNTs and appropriate SDS to the electrolyte, Cu-CNTs composite coating with high CNTs content and uniformly distributed CNTs was successfully prepared by jet electrodeposition.

**Figure 3:**
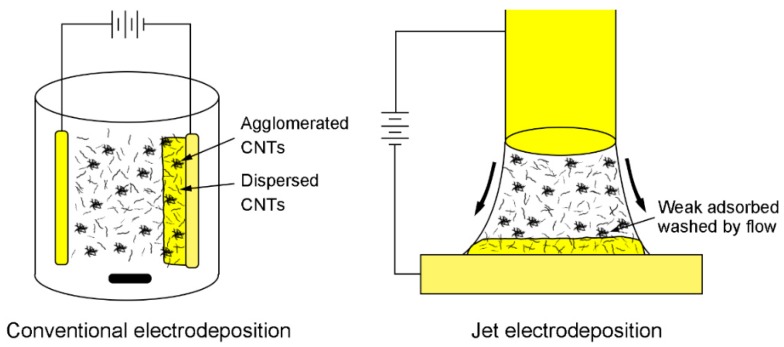
Schematic of conventional electrodeposition and jet electrodeposition.

A schematic of conventional electrodeposition and jet electrodeposition is shown in Figure 3. In conventional electrodeposition, some agglomerated CNTs can be captured by the Cu matrix during the electrodeposition process. The high-speed jet flow plays a positive role in reducing agglomeration in jet electrodeposition. In Guglielmi’s two-step adsorption model [33], the second phases are weakly adsorbed on the cathode surface in the first step. Our experimental results demonstrate that the scouring of high-speed jet flow could effectively reduce CNTs agglomeration by washing CNTs away when they are weakly adsorbed on the cathode surface. In contrast with conventional electrodeposition, more CNTs can reach the cathode surface due to the high speed flow of the electrolyte in jet electrodeposition. Therefore, the probability of CNTs being captured by the Cu matrix increases and the incorporated CNTs content significantly increases.

**Figure 4:**
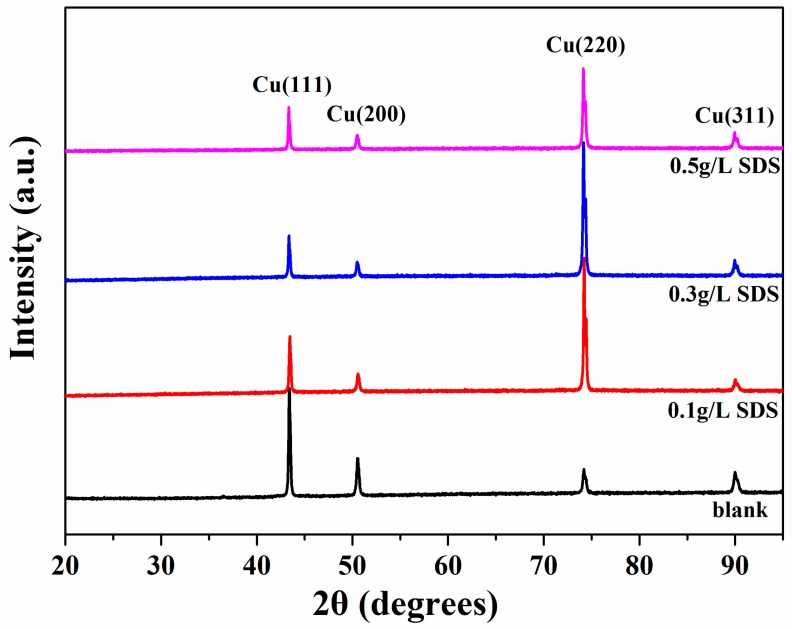
X-ray diffraction (XRD) patterns of the Cu-CNTs composite coatings prepared without SDS and with different SDS concentrations (100 mg/L, 300 mg/L, and 500 mg/L).

XRD patterns of the Cu-CNTs composite coatings are shown in Figure 4. The (111) plane is the preferred orientation of the Cu matrix without SDS additive. When the SDS was added to the electrolyte, the (220) plane became the preferred orientation. These results can be attributed to interfacial energy-driven phenomena [32]. The XRD results showed that the diffraction patterns of the Cu matrix do not change significantly with varying SDS concentration in the electrolyte.

**Figure 5:**
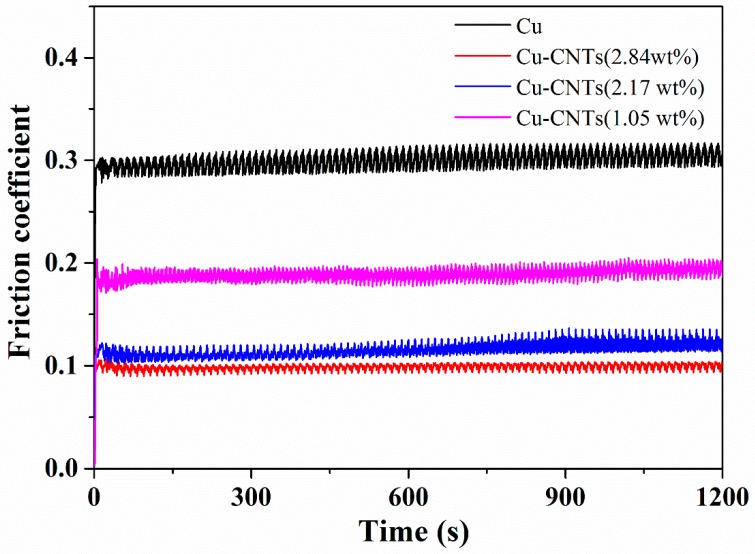
Evolution of friction coefficients of Cu and Cu-CNTs composites over time.

The microhardness and wear test results for the pure Cu and Cu-CNTs composite coatings are shown in Table 2 and Figure 5. We found that the friction coefficient and wear weight loss of the Cu-CNTs composites decreased and microhardness improved with increasing CNTs content. The pure Cu (85 Hv) showed a friction coefficient of 0.28 and wear weight loss of 8.9 × 10^−2^ mg/Nm, which can be attributed to the low microhardness and low plastic deformation resistance. The composite coating with a CNT content of 1.05 wt % (127 Hv) had a friction coefficient of 0.18 and wear weight loss of 4.5 × 10^−2^ mg/Nm. The composite coating with 2.17 wt % CNTs content (147 Hv) had a friction coefficient of 0.12 and wear weight loss of 2.2 × 10^−2^ mg/Nm. When the CNT content was as high as 2.84 wt %, the composite coating (155 Hv) had a coefficient friction of 0.11 and wear weight loss of 1.3 × 10^−2^ mg/Nm. The friction coefficient showed an inverse relationship with microhardness. With increasing microhardness, the friction coefficient of the composite coatings decreased due to the high deformation resistance. The wear weight loss of composite coatings was sensitive to the CNTs content, which can be attributed the self-lubrication property of CNTs. With higher CNT content, more CNTs existed on the friction surface and less Cu matrix was worn away. The composite coating with a 2.17 wt % CNT content had a friction coefficient of 0.12 and the composite coating with 2.84 wt % CNTs had a friction coefficient of 0.11, which can be attributed to the close microhardness values (147 Hv and 155 Hv, respectively) of these two composite coatings. The above two composite coatings showed obvious differences in wear weight loss (2.2 × 10^−2^ mg/Nm and 1.3 × 10^−2^ mg/Nm, respectively), which can be attributed to different CNT contents (2.17 wt % vs. 2.84 wt %, respectively) and the formation of coarse grains on the surface. The coarse grains on the surface of the composite coatings (2.17 wt % CNTs) led to an increase in wear weight loss.

**Figure 6:**
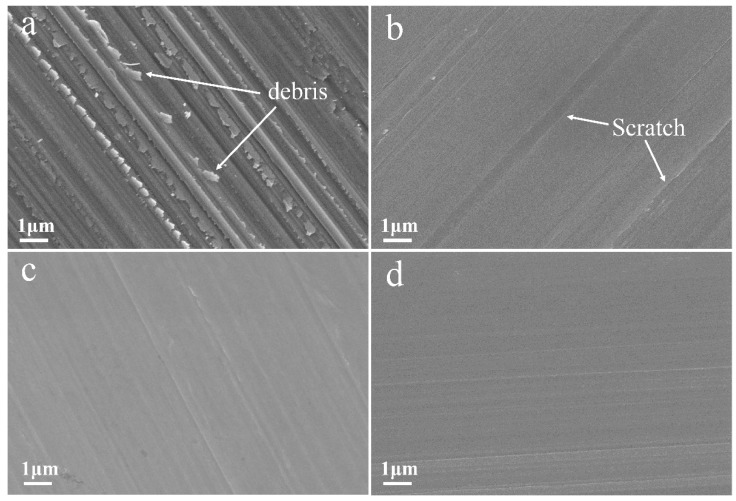
Micrograph of worn surface for (**a**) Cu, (**b**) Cu-CNTs (1.05 wt %), (**c**) Cu-CNTs (2.17 wt %), and (**d**) Cu-CNTs (2.84 wt %).

The SEM morphology of the worn surface of pure Cu is shown in Figure 6a. It can be seen that the wear track of the pure Cu had large pieces of debris and deformation parts. The local stress on the contact surfaces caused plastic deformation, and the atoms on the contact surfaces were stuck by the bonding of the atoms (cold welding). In the subsequent sliding process, the sticking point was cut and fell off to form wear debris. For the composite worn surface shown in Figure 6b,c, the worn surfaces were much smoother due to less plastic deformation on the contact surface [34]. As the soft Cu matrix easily flaked off during the wear process, the CNTs (high content) were exposed and formed the lubricating film on the worn surface [25]. The lubricating film can significantly reduce the adhesive wear between the GCr15 ball and the Cu matrix, which resulted in low wear weight loss. These results are highly consistent with previous works [26,27]. 

## 4. Conclusions

A simple and feasible jet electrodeposition method was used to prepare Cu-CNTs composite coatings. Without any treatment of the CNTs before electrodeposition, we fabricated Cu-CNTs composite coatings with high CNT content and uniformly dispersed CNTs. The wear rates and friction coefficients of these Cu-CNTs composites showed a decreasing trend with increasing CNT content. Due to the self-lubrication properties, the high CNT content formed a lubricating film that effectively reduced the contact between the wear ball and Cu matrix. Therefore, the tribological properties of the Cu-CNTs composite coatings were significantly enhanced. This method provides an effective approach to embedding a one-dimensional reinforcement phase into a metal matrix.

## Figures and Tables

**Table 1:** Zeta potential, Carbon nanotubes (CNT) content and microhardness of the composite coatings without sodium dodecyl sulfate (SDS) and with different SDS concentration in the electrolyte.

SDS Concentration (g/L)	Zeta Potential (mV)	CNT Content (wt %)	Microhardness (Hv)
0	−6.1 ± 2.1	-	-
0.1	−12.8 ± 1.1	1.05	127
0.3	−16.8 ± 1.5	2.84	155
0.5	−24.5 ± 2.4	2.17	147

**Table 2:** Microhardness and wear performance of Cu and Cu-CNTs composite coatings.

CNTs Content (wt %)	Microhardness (Hv)	Friction Coefficient	Wear Weight Loss (mg/Nm)
0	85	0.28	8.9 × 10^−2^
1.05	127	0.18	4.5 × 10^−2^
2.17	147	0.12	2.2 × 10^−2^
2.84	155	0.11	1.3 × 10^−2^
